# Strategies for Monitoring Serum Protein Degradation With an Antibody Array‐Based Technology

**DOI:** 10.1002/jcla.70173

**Published:** 2026-02-13

**Authors:** Yanlin Wang, Min Lang, Wei Huang, Siwei Zhu, Yingqing Mao, Shuhong Luo, Hua Dong, Ruo‐Pan Huang

**Affiliations:** ^1^ Department of Biomedical Engineering, School of Materials Science and Engineering South China University of Technology Guangzhou Guangdong China; ^2^ Raybiotech Co. Ltd., Guangzhou Guangzhou Guangdong China; ^3^ South China Biochip Research Center Guangzhou Guangdong China; ^4^ Zhujiang Hospital of Southern Medical University Guangzhou Guangdong China; ^5^ RayBiotech Inc. Peachtree Corners Georgia USA; ^6^ National Engineering Research Center for Tissue Restoration and Reconstruction (NERC‐TRR) Guangzhou Guangdong China; ^7^ Affiliated Cancer Hospital and Institute of Guangzhou Medical University Guangzhou Guangdong China

**Keywords:** antibody array, biomarkers, linear regression, protein degradation, serum

## Abstract

**Background:**

Human serum is an ideal body fluid for discovering and monitoring biomarkers for disease diagnosis and treatment response. However, intrinsic proteolytic degradation during sample handling may compromise biomarker integrity, which may affect the accuracy of results. To address this issue, we aimed to test the feasibility of using antibody array technology to evaluate the temporal stability of serum proteins at room temperature.

**Methods:**

Concentrations of 480 serological proteins were monitored using antibody arrays in samples from 10 healthy donors incubated at 25°C for 0, 6, 12, 24, and 48 h. Linear regression assessed time‐dependent concentration changes. Physicochemical properties (molecular weight, isoelectric point, instability index, aliphatic index, hydropathicity) of the proteins were analyzed. Enrichment analyses were performed on degraded proteins.

**Results:**

During 48‐h incubation, 201 proteins showed a significant negative linear correlation between concentration and time, among which the concentration of 91 proteins reduced over 20% in the first 6 h. Degraded proteins were significantly associated with lower molecular weight (MW < 40 kDa) but no other physicochemical properties. Enrichment analyses revealed degraded proteins associated with various terms and involved in important signaling pathways, like JAK–STAT, PI3K‐Akt, and MAPK.

**Conclusion:**

Our data demonstrate the feasibility of employing antibody arrays for detecting serum protein degradation. Using this platform, we found some serum proteins with clinical significance rapidly degrade at room temperature, including interleukins (e.g., IL‐1β/IL‐12p40/IL‐17), growth factors (e.g., aFGF, bFGF, and BMP‐2), and chemokines (e.g., I‐309, 6Ckine, and BLC). These may serve as potential biomarkers for assessing human serum sample quality.

## Graphical abstract

This study analyzed the stability of 480 proteins in human serum samples collected from 10 healthy donors and incubated at room temperature for 0–48 h. Antibody array profiling revealed significant time‐dependent degradation of 201 proteins. Subsequent physicochemical and functional enrichment analyses demonstrated that the degraded proteins were associated with specific structural properties and biological pathways. These findings validate the utility of antibody arrays in monitoring proteolytic degradation and suggest potential biomarkers for assessing serum sample quality in laboratory and clinical diagnostics.

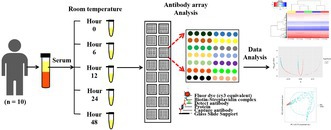

## 1 Introduction

Human blood proteome is of great significance for studying disease physiology and pathology [1]. Human serum and plasma samples have been widely used in biological and clinical studies [2]. Serum is the fluid part of blood that is obtained after blood collection and a proteolytic clotting process followed by centrifugation to remove insoluble content, including cells and fibrin clots [2, 3]. It is believed that serum contains thousands of distinct proteins as well as various small molecules, including salts, lipids, amino acids, and sugars [4]. The major protein constituents of serum include albumin, immunoglobulins, transferrin, haptoglobin, and lipoproteins [4, 5]. In addition to these major constituents, serum also contains many other proteins that are synthesized and secreted, shed, or lost from cells and tissues throughout the body [6, 7]. Serum is an ideal clinical sample that contains an archive of information due to the presence of a variety of proteins released by tissues, thus reflecting the physiological and pathological status of the patient [8, 9]. This protein profile can change rapidly [8]. Moreover, serum is easily accessible, relatively inexpensive, and minimally invasive to collect [10]. Serum protein biomarkers have been widely used in clinical diagnoses, as well as predicting and monitoring therapeutic response in many diseases, including cancer, cardiovascular disease, and hypertension [11, 12, 13, 14].

The serum proteome is vulnerable to proteolytic degradation due to intrinsic peptidase activities that occur during improper sample collection and storage [15]. Protein and peptide fragments resulting from this degradation can lead to inaccurate diagnostic test results, potentially harming the patient [16]. It is widely acknowledged that controlling pre‐analytical factors including sample collection, transportation, handling, processing, and storage is crucial for maintaining sample quality, which can be achieved by implementing strict standardized protocols [1, 17]. However, the effectiveness of these protocols is highly dependent on consistent and correct application. Despite this, compromised sample quality remains an issue due to variations in collection protocols and improper adherence to standardized procedures. Protein degradation, which is not visible to the naked eye, underscores the need for identifying biomarkers that accurately reflect serum quality. To address this challenge, proteomic‐based analyses using mass spectrometry (MS) approaches have been employed. For example, Craft et al. [18] developed a liquid chromatography/matrix‐assisted laser desorption/ionization (LC‐MALDI) method to assess relative peptide stability. Yi et al. [15] investigated plasma and serum sample stability by monitoring specific peptides using matrix‐assisted laser desorption/ionization time‐of‐flight mass spectrometry (MALDI‐TOF‐MS). Their findings revealed intrinsic proteolytic degradation in both serum and plasma samples at room temperature [15]. Rezeli et al. [1] explored the stability of human plasma samples by incorporating stable isotope‐labeled peptides as internal standards and monitoring their degradation under various storage conditions using MALDI‐MS and LC–MS platforms. The study demonstrated that isotope‐labeled peptides remained stable in plasma at −20°C and −80°C over a two‐month period, while proteolytic degradation occurred at room temperature [1]. However, the limitations in sensitivity inherent to MS‐based methods pose a significant challenge for multiplexed detection of low‐abundance protein biomarkers in clinical samples [19, 20]. Crucially, low‐abundance serum proteins, such as the interleukins, tumor necrosis factors, and interferons, play a critical role in the etiopathogenesis of diseases and serve as important markers in the laboratory tests of some diseases [21]. Therefore, it is very important to directly monitor the degradation of low‐abundance serum proteins with non‐MS‐based methods to ensure the accuracy of disease diagnosis.

Antibody arrays present a highly suitable alternative for this purpose. This technology employs various antibodies spotted onto array surfaces, which capture specific antigens and allow for the quantitative measurement of their concentrations, including low‐abundance proteins [22, 23]. These sandwich‐based arrays use a pair of two different antibodies to detect one protein: one immobilized antibody to capture the protein and one detection antibody binding to a separate epitope [22, 24]. Thus, protein detection relies on the presence and availability of two distinct epitopes for binding antibody. Proteolytic degradation can disrupt the binding of one or both antibodies by cleaving epitopes, thereby leading to a measurable reduction in detection signal, which can serve as an indicator of degradation. Antibody arrays are well‐established in biomedical research and clinical diagnostics as they enable the simultaneous detection of hundreds to thousands of proteins using minimal sample volume [22, 23, 24, 25, 26]. They are compatible with various biological sample types, including serum, plasma, urine, and tissue [27]. Therefore, to directly address the critical need for monitoring the ex vivo degradation of clinically relevant low‐abundance serum proteins, we employed comprehensive glass‐slide‐based antibody arrays. This study aimed to investigate the temporal degradation profile of 480 key low‐abundance proteins, predominantly cytokines, in human serum stored at room temperature for up to 48 h using this high‐throughput immunoassay platform.

## 2 Materials and Methods

### 2.1 Collection and Preparation of Serum Samples

Human whole blood was obtained by venipuncture from the median cubital veins of 10 healthy donors, consisting of 5 men and 5 women with a mean age of 26.9 years (ranging from 23 to 30 years). This age range was intentionally selected to minimize age‐related variations in serum protein profiles, as aging significantly alters protein expression [28]. The human serum samples were collected at the Sun Yat‐sen Memorial Hospital. This study was approved by the institutional ethics committee of the Sun Yat‐sen Memorial Hospital, Sun Yat‐sen University (Approval Number: [2017] Lun Shen Fu No. 06). Naturally occurring proteases are present in serum [15], and to this end, their activity was assessed without the addition of protease or enzyme inhibitors at any stage of blood collection, processing, or storage. The blood samples were allowed to clot for 60 min, then centrifuged at 3000 *g* for 15 min at room temperature to isolate serum samples. The serum was transferred into clean and sterilized tubes, aliquoted, and frozen at −80°C until use [29]. The sample collection and preservation process was completed within 4 h after blood draw. For time‐course experiments, these aliquots were completely thawed at 4°C then incubated for 0, 6, 12, 24, and 48 h at room temperature (25°C) while being protected from light.

### 2.2 Antibody Array Analysis

Two series of RayBio Human Antibody Arrays (QAH‐CAA‐440 and QAH‐CYT‐11; RayBiotech, Peachtree Corners, GA, USA) were used to measure the concentration of 480 soluble proteins in the serum samples (Table S1 for the target list). The assay was carried out in strict accordance with the manufacturer's instructions [30]. Briefly, serum samples were diluted 2‐fold, which was determined through a preliminary trial. The arrays were blocked and then incubated with the diluted samples overnight at 4°C with gentle shaking. Subsequently, the arrays were washed and exposed to biotin‐conjugated detection antibodies for 2 h at room temperature with gentle shaking. Afterward, the arrays were washed again and incubated with Cy3‐equivalent dye‐conjugated streptavidin for 1 h at room temperature with gentle shaking. Finally, the arrays were washed and scanned using an InnoScan 300 Microarray Scanner (Innopsys, Carbonne, France), and the raw signal data were extracted using MapPix 6.0 software. For data normalization and concentration calculation, the signals were processed using the Q‐Analyzer Software from RayBiotech. Standard curves were generated for each target protein. Intra‐array normalization was performed using two positive control spots in each well, and inter‐array normalization was achieved using the negative control and the Standard 3 wells [30], both applied automatically by the software's built‐in algorithms.

### 2.3 Analysis of Protein Physicochemical Properties

Various physicochemical properties of the measured proteins were evaluated, including molecular weight (MW), theoretical isoelectric point (pI), instability index [31], aliphatic index [32], and grand average of hydropathicity (GRAVY) [33]. This analysis was conducted using the ProtParam tool online server (accessed: December 2023), which can be accessed at http://web.expasy.org/protparam/ [34, 35].

### 2.4 Bioinformatics Analysis

Gene Ontology (GO) and Kyoto Encyclopedia of Genes and Genomes (KEGG) enrichment analyses were conducted on the 201 proteins that exhibited significantly negative linear correlations between protein concentration and time. Functions and pathways that showed significant enrichment had *p* < 0.05. Principal component analysis (PCA) was performed using the R package “ggbiplot”, hierarchical clustering analysis with the R package “gplots”, GO analysis with the R package “org.Hs.eg.db” and “clusterProfiler”, and KEGG enrichment analysis with the R package “clusterProfiler”, all using the open‐source R software. The STRING database version 12.0 (https://string‐db.org/; accessed: October 2023) was used to predict the significantly enriched protein structures of the serum degradation protein biomarkers.

### 2.5 Statistical Analysis

Statistical analyses were conducted using R software version 3.5.1 (http://www.R‐project.org), GraphPad Prism version 6.0 (GraphPad Software, San Diego, CA, USA), and SPSS 22.0 software (SPSS, Chicago, IL, USA). Linear regression analysis was used to assess the relationship between changes in protein concentration over time based on antibody array data. Following the standardization (*z*‐score) of all protein concentration data, linear regression analysis was performed to determine the regression coefficient (*b*) and *p*‐value. The variable “time” was used with or without a logarithmic transformation of ln (*x* + 0.1), respectively. Chi‐squared tests and Wilcoxon Rank Sum tests were employed to identify differences in the physicochemical properties among the groups under investigation. Statistical significance was considered as *p* < 0.05 in all instances.

## 3 Results

### 3.1 Identification of Serum Protein Degradation Biomarkers With an Antibody Array

**FIGURE 1:**
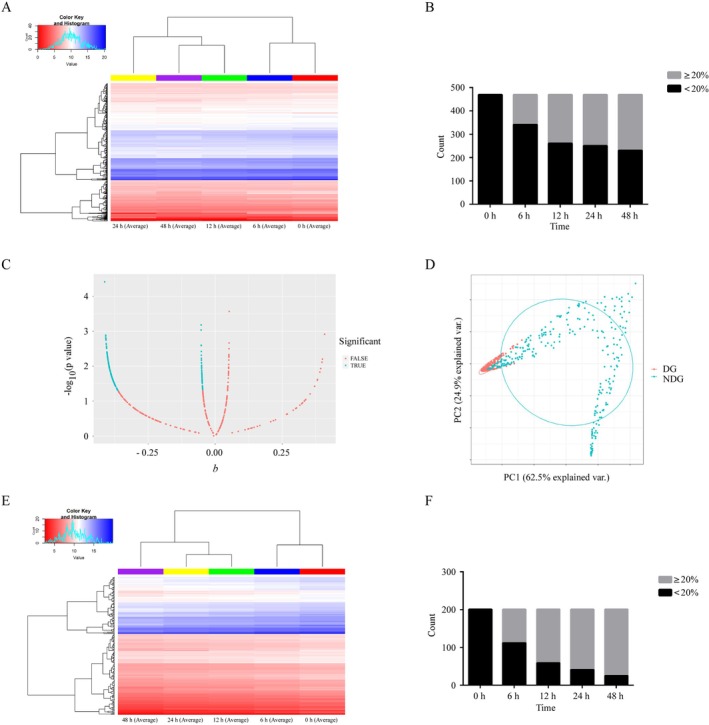
Concentration changes of 470 serum proteins analyzed over time using an antibody array. (A) The heatmap shows the changes in the concentration of 470 serum proteins over time at room temperature. The colors represent log_10_ transformed protein concentrations, with red indicating low concentrations and blue representing high concentrations. (B) Serum protein concentrations decreased by 20% in all 470 proteins at each time point. (C) Linear regression analysis of serum protein concentrations and time at room temperature. The *X*‐axis: The regression coefficient (*b*) and the *Y*‐axis: The *p‐*value (−log_10_ transformed) of proteins. (D) A PCA map was created for the DG and NDG proteins. (E) Hierarchical clustering of the DG proteins. Red = low concentrations, blue = high concentrations. (F) Serum protein concentrations decreased by 20% in the DG at each time point. DG, degraded proteins group; NDG, non‐degraded proteins group.

Out of the 480 proteins analyzed by antibody arrays, 470 proteins remained for further analysis after standardizing the array data and eliminating the proteins that had a concentration of 0 pg/mL at any of the measured time points (Tables S1 and S2). Figure 1A depicts the heat map of the concentration profiles of these 470 proteins at different time points. The results revealed that the concentrations of these proteins declined over time when the serum was stored at room temperature. After 6, 12, 24, and 48 h, the concentration of 128, 209, 219, and 239 proteins had decreased by more than 20% compared to the 0‐h mark, respectively (Figure 1B). At 48 h, the concentration of 151 proteins decreased by 20%–50%, and the concentration of 88 proteins decreased by 50%–90%.

Next, a linear regression analysis was conducted to evaluate the association between changes in protein concentration over time. Figure 1C shows a volcano plot generated based on the beta and *p*‐values of the 470 proteins. Among these, 201 proteins exhibited a significantly negative linear correlation between concentration and time‐course changes (*beta* < 0 and *p* < 0.05; Table S2), categorizing them into the “degraded protein group” (DG; Table 1). The remaining 269 proteins were classified as the “non‐degraded protein group” (NDG). Using the *beta* and *p*‐values for each protein, PCA was utilized to identify differentially expressed serum proteins among the groups (Figure 1D). Hierarchical clustering analysis further segregated the 201 proteins into two primary clusters (Figure 1E). Within the DG, the concentration of 91, 142, 160, and 176 serum proteins decreased by more than 20% after 6, 12, 24, and 48 h of storage, respectively (Figure 1F). Notably, proteins exhibiting significant concentration decrease over time encompass multiple major families, including interleukins, cytokine receptors, chemokines, growth factors, enzymes and inhibitors, adhesion molecules, hormones, TNF/TNFR superfamily, immunoglobulin superfamily, etc., which are involved in complex physiological functions. However, similar protein families were also observed in the NDG group.

**TABLE 1:** Two hundred and one serum proteins in degraded protein group (*b* < 0 and *p* < 0.05).

Protein name	Protein ID	Protein name	Protein ID	Protein name	Protein ID
4‐1BB	Q07011	FGF‐17	O60258	LIGHT	O43557
6ckine	O00585	FGF‐19	O95750	LOX‐1	P78380
ADAM8	P78325	FGF‐21	Q9NSA1	LRP‐6	O75581
ADAM9	Q13443	FGF‐4	P08620	L‐Selectin	P14151
aFGF	P05230	FGF‐6	P10767	Lymphotactin	P47992
AFP	P02771	FGF‐7	P21781	LYVE‐1	Q9Y5Y7
AgRP	O00253	FLRG	O95633	Marapsin	Q9BQR3
AMICA	Q86YT9	Follistatin	P19883	MCP‐2	P80075
ANG‐1	Q15389	FOLR1	P15328	MCP‐3	P80098
ANG‐2	O15123	Fractalkine	P78423	MCP‐4	Q99616
Angiotensinogen	P01019	Furin	P09958	MDM2	Q00987
APRIL	O75888	Galectin‐1	P09382	Mer	Q12866
AR	P15514	Galectin‐2	P05162	Midkine	P21741
B2M	P61769	Galectin‐3	P17931	MIP‐3a	P78556
B7‐H1	Q9NZQ7	Galectin‐8	O00214	MMP‐7	P09237
B7‐H3	Q5ZPR3	GCP‐2	P80162	MPIF‐1	P55773
bFGF	P09038	G‐CSF R	Q99062	MSP	P26927
BLAME	Q9P0V8	GITR L	Q9UNG2	Nectin‐1	Q15223
BLC	O43927	Glypican 1	P35052	Neprilysin	P08473
BMP‐2	P12643	Glypican 5	P78333	NGF R	P08138
BMP‐5	P22003	gp130	P40189	Nidogen‐1	P14543
BMP‐7	P18075	GRO	P09341	NKp30	O14931
BMP‐9	Q9UK05	HAI‐2	O43291	Notch‐1	P46531
BMPR‐IB	O00238	HCC‐1	Q16627	NSE	P09104
b‐NGF	P01138	HGF	P14210	NT‐4	P34130
BTC	P35070	I‐309	P22362	OPG	O00300
CA19‐9	Q969X2	ICOS	Q9Y6W8	PAI‐1	P05121
CA9	Q16790	IFNab R2	P48551	PD‐1	Q15116
Cadherin‐11	P55287	IGF‐2R	P11717	PECAM‐1	P16284
Cadherin‐13	P55290	IL‐1 F10	Q8WWZ1	Pentraxin 3	P26022
Cadherin‐4	P55283	IL‐1 F8	Q9NZH7	Persephin	O60542
CCL28	Q9NRJ3	IL‐1 R3	Q9NPH3	Pref‐1	P80370
CD200	P41217	IL‐1 R6	Q9HB29	Prolactin	P01236
CD229	Q9HBG7	IL‐1 RII	P27930	Renin	P00797
CD27	P26842	IL‐10 Ra	Q13651	RGM‐B	Q6NW40
CD30	P28908	IL‐10 Rb	Q08334	S100A8	P05109
CD48	P09326	IL‐11	P20809	SCF	P21583
CD58	P19256	IL‐12p40	P29460	SDF‐1a	P48061
CD6	P30203	IL‐17	Q16552	Semaphorin 7A	O75326
CD84	Q9UIB8	IL‐17B	Q9UHF5	sFRP‐3	Q92765
CD97	P48960	IL‐17E	Q9H293	Siglec‐10	Q96LC7
CEACAM‐5	P06731	IL‐18	Q14116	SLAM	Q13291
CF XIV	P04070	IL‐18 BPa	O95998	ST2	Q01638
Chemerin	Q99969	IL‐1b	P01584	Syndecan‐3	O75056
Ck beta 8–1	P55773‐2	IL‐2 Ra	P01589	TACI	O14836
Clusterin	P10909	IL‐2 Rb	P14784	TECK	O15444
Common beta Chain	P32927	IL‐2 Rg	P31785	Testican 2	Q92563
CTLA4	P16410	IL‐21	Q9HBE4	TF	P13726
CXCL16	Q9H2A7	IL‐22 R alpha 1	Q8N6P7	TGF‐b1	P01137
Cystatin A	P01040	IL‐23 R	Q5VWK5	TGF‐b2	P61812
DcR3	O95407	IL‐24	Q13007	TGF‐b3	P10600
Desmoglein 2	Q14126	IL‐28A	Q8IZJ0	Thrombomodulin	P07204
Desmoglein‐3	P32926	IL‐29	Q8IU54	Thrombospondin‐5	P49747
Dkk‐4	Q9UBT3	IL‐3	P08700	TIM‐3	Q8TDQ0
DR3	Q93038	IL‐32 alpha	P24001	TLR2	O60603
Dtk	Q06418	IL‐33	O95760	TLR4	O00206
EDA‐A2	Q92838	IL‐5 Ra	Q01344	TRAIL	P50591
EGF R	P00533	IL‐9	P15248	TRAIL R1	O00220
EG‐VEGF	P58294	Integrin alpha 5	P08648	TRAIL R4	Q9UBN6
ENA‐78	P42830	I‐TAC	O14625	Transferrin	P02787
Endoglin	P17813	JAM‐A	Q9Y624	Troponin I	P48788
Eotaxin	P51671	JAM‐B	P57087	TSH	P01222
Eotaxin‐3	Q9Y258	Kallikrein 14	Q9P0G3	ULBP‐1	Q9BZM6
EphB6	O15197	Kallikrein 5	Q9Y337	VE‐Cadherin	P33151
Epo R	P19235	LAP (TGFb1)	P01137	VEGF	P15692
ESAM	Q96AP7	Leptin	P41159	VEGF R3	P35916
FABP2	P12104	LIF	P15018	WIF‐1	Q9Y5W5

### 3.2 Physicochemical Characteristics of the Proteins

**FIGURE 2:**
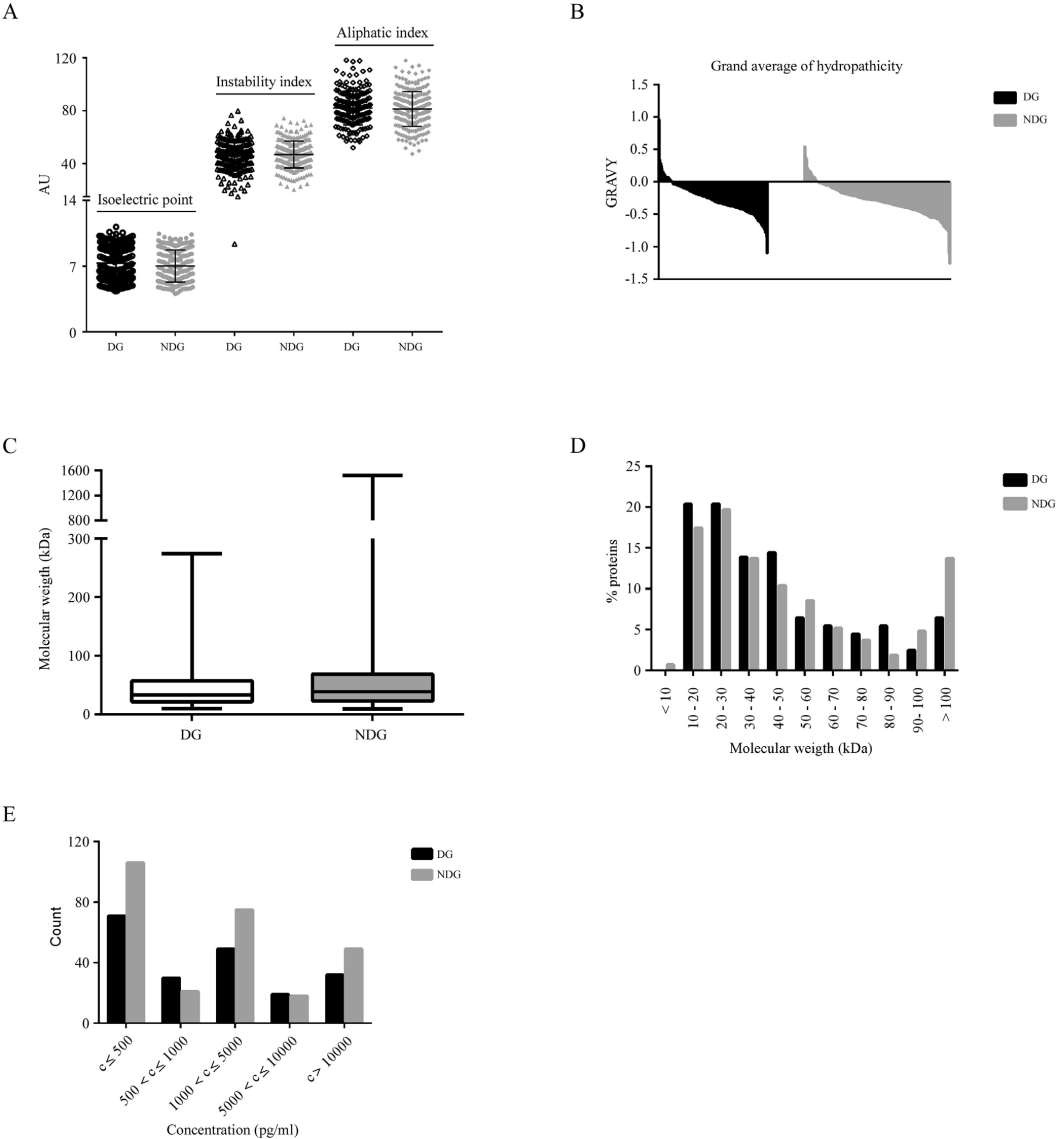
Physicochemical properties of serum proteins. (A) Scatter plots of the isoelectric point, instability index, and aliphatic index in the DG (black) and NDG (gray) proteins. No statistically significant difference was found between the groups. The black horizontal lines represent the average, while the error bars represent the standard deviations. (B) Plots of the GRAVY values of the DG (black) and NDG (gray) proteins. (C) Box plot of the molecular weights from the DG (black) and NDG (gray) proteins. (D) Analysis of the molecular weights of the DG (black) and NDG (gray) proteins. Large proteins (> 90 kDa) were more often found in the NDG group. (E) Concentration distribution of DG (black) and NDG (gray) proteins at 0 h. AU, arbitrary units; DG, degraded proteins group; GRAVY, grand average of hydropathicity; NDG, non‐degraded proteins group.

To gain a deeper understanding of the specific features of both DG and NDG, we analyzed various physicochemical properties of the 470 proteins, including theoretical pI, MW, instability index, aliphatic index, GRAVY, and concentration (Figure 2 and Table S3). We also investigated the correlation between the physicochemical properties of the proteins and their degradation (Table 2).

**TABLE 2:** Associations between serum proteins and physicochemical properties.

Characteristics	DG	NDG	Total (% 0f 470)	*χ* ^2^	*p*
PI					
≤ 7.0	98	144	242 (51.5%)	1.050	0.305
> 7.0	103	125	228 (48.5%)		
Grand average of hydropathicity (GRAVY)					
GRAVY ≤ 0	175	245	420 (89.4%)	0.743	0.389
GRAVY > 0	26	24	50 (10.6%)		
Concentration (pg/mL)					
c ≤ 500	71	106	177 (37.7%)	7.882	0.096
500 < c ≤ 1000	30	21	51 (10.8%)		
1000 < c ≤ 5000	49	75	124 (26.4%)		
5000 < c ≤ 10,000	19	18	37 (7.9%)		
c > 10,000	32	49	81 (17.2%)		
Instability index					
Stable	52	67	119 (25.3%)	0.056	0.812
Unstable	149	202	351 (74.7%)		
Aliphatic index					
AI ≤ 100	185	245	430 (91.5%)	0.137	0.712
AI > 100	16	24	40 (8.5%)		
Molecular weight (MW; kDa)					
M ≤ 20	49	41	90 (19.1%)	9.057	0.029*
20 < M ≤ 40	90	69	159 (33.8%)		
40 < M ≤ 90	80	73	153 (32.6%)		
M > 90	50	18	68 (14.5%)		

Abbreviations: DG, degraded protein group; NDG, non‐degraded protein group.

* Significant correlation between MW and degradation of the proteins.

The pI is the pH at which the protein's net charge is zero [36, 37]. The pI values of the 201 proteins in DG ranged from 4.36 to 11.18, with an average of 7.33 (Figure 2A). For 98 proteins, the pI was alkaline. For the remaining 103 proteins, the pI was acidic. The pI values of NDG proteins ranged from 4.06 to 10.46, with an average of 7.03 (Figure 2A). There were no significant differences in protein pI between the NDG and DG groups (*p* > 0.05).

The instability index reflects the stability of a protein in vitro, with a value of “40” serving as the threshold between a stable protein (< 40) and an unstable protein (> 40) [32, 36, 37]. Increased instabilities lead to a higher instability index. The instability index of DG proteins ranged from 9.37 to 79.88 (Figure 2A). 52 DG proteins had an instability index < 40, while 149 proteins had an instability index > 40. The instability index of NDG proteins ranged from 20.36 to 74.48 (Figure 2A). There were no significant differences in the instability index between the NDG and DG groups (*p* > 0.05).

The aliphatic index, defined as the relative volume of a protein occupied by aliphatic side chains (alanine, valine, isoleucine, and leucine), may help increase the thermal stability of globular proteins [31, 34, 35]. In other words, the higher the aliphatic index, the more stable a protein is across a broad range of temperatures. The aliphatic index for the proteins in DG ranged from 51.96 to 118.20, while the aliphatic index for the proteins in NDG ranged from 74.33 to 117.92 (Figure 2A). No significant differences in the aliphatic index between the NDG and DG groups were found (*p* > 0.05).

The GRAVY value for a peptide or protein is calculated as the sum of hydropathy values of all the amino acids divided by the number of residues in the sequence [34, 36, 37]. A low GRAVY value indicates that a protein is hydrophilic. GRAVY indices of the proteins in DG ranged from −1.098 to 0.965, while NDG proteins ranged from −1.258 to 0.551. Figure 2B shows the rank of 201 DG proteins from the most hydrophobic (with the highest positive GRAVY score) to the most hydrophilic (with the lowest negative GRAVY score). Although NDG had more proteins with positive GRAVY scores (12.9%) than DG (9.7%), no significant differences in GRAVY between the two groups were found (*p* > 0.05).

The distribution of DG and NDG proteins spanned similar concentration ranges. At low concentrations (≤ 1000 pg/mL), there were 127 proteins in the NDG group and 101 in the DG group, with NDG proteins accounting for 55.7% in this range. This percentage is similar to that in the > 1000 pg/mL range (142/242, 58.7%), aligning with the overall NDG proportion in the 470 proteins (57.2%). Accordingly, statistical analysis revealed no significant association between protein degradation and concentration (*p* > 0.05; Table 2).

Finally, we examined the MWs of the proteins in the NDG and DG groups. The 470 proteins exhibited a wide MW distribution: 19.1% < 20 kDa, 33.8% at 20–40 kDa, 32.6% at 40–90 kDa, and 14.5% > 90 kDa (Table 2). The DG proteins exhibited MWs ranging from 10.103 to 274.375 kDa with an average MW of 46.015 kDa, while the NDG proteins exhibited MWs ranging from 9.849 to 1519.175 kDa with an average MW of 58.987 kDa (Figure 2C). Notably, more than 50% of proteins in the DG had MWs < 40 kDa, while proteins with MWs > 90 kDa were more commonly found in the NDG (Figure 2D). Among DG proteins having MWs < 40 kDa, the top four families are chemokines (over 30% proteins), growth factors, interleukins, and TNF/TNFRSF, respectively. The degradation of proteins was significantly associated with their MWs (*p* = 0.029; Table 2).

### 3.3 Bio‐Informatics Analysis of Degradation Proteins

**FIGURE 3:**
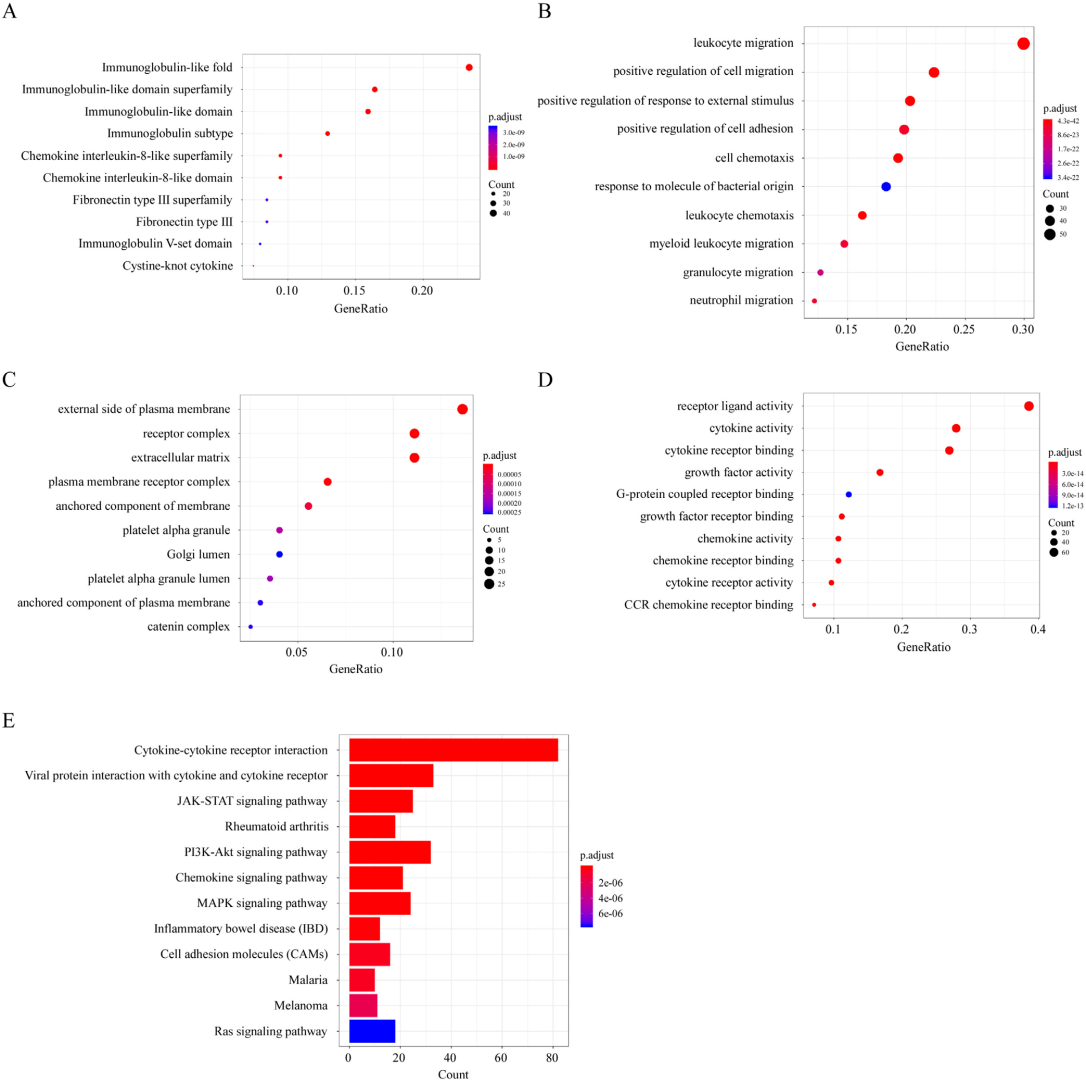
Bioinformatics analysis of degraded proteins. (A) Domain enrichment analysis of the 201 degraded proteins. (B–D) Gene Ontology enrichment analysis of the degraded proteins in terms of (B) biological processes, (C) molecular functions, and (D) cellular components. (E) Kyoto Encyclopedia of Genes and Genomes enrichment analysis of pathways significantly enriched in the 201 degraded proteins.

Finally, we conducted a more in‐depth analysis of the domains, GO terms, and KEGG pathway functions of the 201 proteins that were identified as DG to understand the biological functions the degraded proteins were involved in (Figure 3 and Table S4). Domain analysis revealed that the proteins were associated with 87 significant functional terms (*p* < 0.05). As shown in Figure 3A, the top three significantly enriched domain terms included immunoglobulin‐like fold (47 proteins), immunoglobulin‐like domain super family (33 proteins), and immunoglobulin‐like domain (32 proteins). GO enrichment analysis indicated that the degraded proteins were significantly (*p* < 0.05) involved in various functional terms within the categories of biological process, molecular function, and cellular component. Figure 3B–D show the top 10 significantly enriched GO terms. KEGG pathway enrichment analysis revealed that the proteins in DG were significantly (*p* < 0.05) linked to 38 signaling pathways, such as cytokine‐cytokine receptor interaction signaling pathways (82 proteins), JAK–STAT signaling pathway (25 proteins), PI3K‐Akt signaling pathway (32 proteins), and rheumatoid arthritis (18 proteins; Figure 3E).

## 4 Discussion

Human serum is an ideal biological sample for evaluating various diseases since it contains a variety of proteins released by tissues and the process of sample collection is minimally invasive [8]. However, the natural presence of proteases in blood leads to rapid degradation of serum proteins, complicating their use as reliable biomarkers and significantly impacting biomarker discovery studies [15, 38]. While MS offers high‐throughput screening capabilities for protein detection, its limitations in reproducibility and sensitivity hinder accurate detection of low‐abundance proteins in complex biofluids [20].

In this study, we employed sandwich‐based antibody arrays to simultaneously measure the concentration of 480 proteins in serum samples. This antibody array technology demonstrates distinct advantages for such an investigation. It combines the high‐throughput capacity necessary for broad‐spectrum screening with the high sensitivity required to detect low‐abundance proteins like cytokines, growth factors, and enzymes [20, 30]. Moreover, the platform is strategically designed to manage the broad dynamic range of protein concentrations in serum, which spans over 10 orders of magnitude [23]. The detection system for each individual target on the array is pre‐calibrated to cover 4–5 orders of magnitude, with its working range optimized to match the expected concentration of the target in serum [23, 39]. Proteins with incompatible detection ranges are distributed across specialized sub‐arrays, enabling simultaneous quantification of targets with vastly different abundances.

Critically, antibody arrays provide high reproducibility, with documented intra‐assay coefficients of variation of 7%–10% [39, 40], offering a distinct advantage compared to MS. The antibody arrays successfully quantified 470 of the 480 targeted proteins, demonstrating broad applicability and capacity to handle the complexity of the human serum proteome. This high success rate underscores the potential of antibody arrays for large‐scale, parallel analysis of proteins, including those present at low abundance which are often challenging to detect with other methods [23, 30]. Meanwhile, the concentrations of well‐characterized cytokines, such as IL‐6 and TNF‐α, remained consistent with previous studies, which have documented their relative stability under multiple storage conditions [41, 42]. The alignment with these established findings may reflect the reliability of quantitative antibody arrays for analyzing protein degradation.

Our study expands the landscape of degradation‐susceptible serum proteins by identifying 201 specific proteins that undergo significant degradation under room temperature storage conditions. Notably, the degraded proteins encompass numerous major families, including many with significant biological importance. Particularly noteworthy examples include several critical interleukins (e.g., IL‐1β, IL‐12p40, and IL‐17), FGFs (e.g., aFGF, bFGF, and FGF‐6), TGF‐β Superfamily (e.g., BMP‐2 and BMP‐9), and chemokines (e.g., I‐309, ENA‐78, and BLC). These proteins represent common clinical biomarkers, while their concentrations decrease by more than 20% after merely 6 h at room temperature. Consequently, they emerge as primary candidates for serving as potential indicators of pre‐analytical sample degradation status. The bioinformatics analysis provided additional clarity on the biological functions and pathways related to the degraded proteins. This discovery highlights the significance of including protein stability in research focused on these pathways, as degradation may result in the absence of crucial functional information.

The physicochemical analysis revealed that proteins with lower MWs (< 40 kDa) were more prone to degradation, while larger proteins (> 90 kDa) were more stable. This observation is consistent with previous studies suggesting that smaller proteins may be more susceptible to proteolytic degradation [43]. The increased susceptibility of smaller proteins to hydrolysis may be attributed to their simpler spatial structures and larger specific surface area, which facilitate protease binding [44]. The lack of correlation with other physicochemical properties suggests that in the complex proteolytic environment of serum, a single intrinsic factor is unlikely to determine a protein's fate. Instead, the stability of proteins is likely regulated in a complex manner by multiple factors, including post‐translational modifications and interactions with other proteins that can shield protease cleavage sites [45, 46].

To contextualize our findings, we note that current studies on the degradation of low‐abundance proteins predominantly employ enzyme‐linked immunosorbent assay or cytometric bead array, which are limited to the detection of dozens of inflammatory factors and chemokines. This results in a significant knowledge gap in the study of other proteins [41]. For example, previous studies using multiplex bead‐based assays or immunoassay panels have quantified only a narrow range of soluble proteins—such as IL‐6, VEGF, and PDGF‐BB—covering less than 20 targets per analysis [47, 48, 49]. Although highly sensitive, these approaches offer only a restricted view of the proteome, focusing largely on cytokines, chemokines, and growth factors. In contrast, our study analyzes 480 proteins, greatly expanding the scope and providing a broader perspective on protein degradation across multiple functional families, thereby addressing a key gap in current knowledge.

Furthermore, the study of pre‐analytical factors on serum or plasma protein stability has yielded conflicting results across the literature, largely due to variations in methodologies, experimental conditions, and the specific analytes measured. One study reported that while many cytokines were stable in unprocessed EDTA blood stored at 4°C for 24 h [47], IL‐6 and TNF‐α were unstable in heparinized plasma at room temperature [49]. Another investigation using a multiplex assay found that IL‐6 and IL‐17A levels declined after just 3 h of pre‐centrifugation delay at room temperature, and IFN‐γ level reduced within 24 h after centrifugation in plasma when keeping at room temperature [46]. In contrast, a comprehensive review noted that IL‐6, TNF‐α, and VEGF are generally stable at 4°C for extended periods [41]. These discrepancies underscore the challenge of establishing universal stability guidelines and highlight the value of large‐scale, systematic studies to create a more comprehensive reference map.

This study has several limitations that should be taken into consideration. First, the analysis was conducted using serum samples from only 10 healthy donors, which may restrict the applicability of the findings to larger populations, especially those with specific diseases or physical conditions. Second, the experiments assessed protein degradation over a 48‐h period at room temperature, failing to account for potential long‐term degradation effects. Third, protein degradation was solely examined through antibody arrays, without cross‐comparison using other methodologies such as MS‐based techniques. Fourth, the analysis of the relationship between serum protein physicochemical properties and degradation in this study was preliminary. The findings based on the five physicochemical parameters are insufficient to elucidate the potential causes of protein degradation. Additionally, the study focused primarily on secreted cytokines and soluble factors among 480 proteins examined, limiting insights into the stability of other protein types. Finally, despite suggesting their potential usefulness, the study did not empirically test the protective effects of protease inhibitors on protein stability.

In conclusion, this study emphasizes the importance of understanding the factors that influence serum protein stability, especially in the context of biomarker discovery and clinical diagnostics. Additionally, it provides evidence that antibody arrays may be a reliable multiplex detection technique for assessing proteolytic degradation of serum biomarkers, indicating its potential usefulness in pre‐analytical quality control for laboratory or clinical serum sample testing. Future research should concentrate on developing strategies to reduce protein degradation, such as optimizing storage conditions or using protease inhibitors, to ensure the reliability of serum‐based biomarkers. Moreover, further investigation into the structural and functional characteristics of proteins that are susceptible to degradation could offer deeper insights into their roles in disease mechanisms and potential therapeutic targets.

## Author Contributions

Conceptualization, Y.M., S.L., and R.‐P.H.; data curation, Y.W.; formal analysis, M.L. and W.H.; funding acquisition, S.L. and R.‐P.H.; investigation, M.L. and S.Z.; methodology, Y.M. and R.‐P.H.; project administration, S.L. and H.D.; resources, S.Z. and R.‐P.H.; software, Y.W. and W.H.; supervision, R.‐P.H.; validation, Y.M. and H.D.; visualization, Y.W. and M.L.; writing – original draft, Y.W. and W.H.; writing – review and editing, Y.W., S.L., and R.‐P.H. All authors have read and agreed to the published version of the manuscript.

## Funding

We would like to express our thanks for the support of Guangzhou 2024 Annual Special Project on Agricultural and Social Development Science and Technology (2024B03J1332, 2024B03J1249), and National Key R&D Program of China (2024YFA1307601, 2024YFA1307602, 2024YFA1307603).

## Ethics Statement

This study was conducted according to the guidelines of the Declaration of Helsinki, and approved by the institutional ethics committee of the Sun Yat‐sen Memorial Hospital, Sun Yat‐sen University (Approval Number: [2017] Lun Shen Fu No. 06).

## Conflicts of Interest

Yanlin Wang, Wei Huang, Siwei Zhu, Yingqing Mao, Shuhong Luo, and Ruo‐Pan Huang are the employees of RayBiotech Inc. Ruo‐Pan Huang has a financial stake in RayBiotech Life as its founder and president. The other authors declare no conflicts of interest.

## Supporting information


**Table S1:** Concentrations of 480 serum proteins analyzed over time using antibody arrays.

**Table S2:** Results of regression analysis and the top 4 principal components of 470 proteins.

**Table S3:** Physicochemical properties of 470 serum proteins determined by ProtParam server.

**Table S4:** Domain, GO, and KEGG enrichment analysis of DG proteins.

## Data Availability

The original contributions presented in this study are included in the article/Supporting Information. Further inquiries can be directed to the corresponding author(s).
